# The role of neuroticism in self-harm and suicidal ideation: results from two UK population-based cohorts

**DOI:** 10.1007/s00127-019-01725-7

**Published:** 2019-05-23

**Authors:** Jonathan D. Hafferty, L. B. Navrady, M. J. Adams, D. M. Howard, A. I. Campbell, H. C. Whalley, S. M. Lawrie, K. K. Nicodemus, D. J. Porteous, I. J. Deary, A. M. McIntosh

**Affiliations:** 1 Division of Psychiatry, Kennedy Tower, Royal Edinburgh Hospital, University of Edinburgh, Edinburgh, EH10 5HF UK; 2 Centre for Genomic and Experimental Medicine, Institute of Genetics and Molecular Medicine, Western General Hospital, University of Edinburgh, Edinburgh, UK; 3 Centre for Cognitive Ageing and Cognitive Epidemiology, University of Edinburgh, Edinburgh, UK

**Keywords:** Neuroticism, Self-harm, Record-linkage, Coping, Ideation

## Abstract

**Background:**

Self-harm is common, debilitating and associated with completed suicide and increased all-cause mortality, but there is uncertainty about its causal risk factors, limiting risk assessment and effective management. Neuroticism is a stable personality trait associated with self-harm and suicidal ideation, and correlated with coping styles, but its value as an independent predictor of these outcomes is disputed.

**Methods:**

Prior history of hospital-treated self-harm was obtained by record-linkage to administrative health data in Generation Scotland:Scottish Family Health Study (*N* = 15,798; self-harm cases = 339) and by a self-report variable in UK Biobank (*N* = 35,227; self-harm cases = 772). Neuroticism in both cohorts was measured using the Eysenck Personality Questionnaire-Short Form. Associations of neuroticism with self-harm were tested using multivariable regression following adjustment for age, sex, cognitive ability, educational attainment, socioeconomic deprivation, and relationship status. A subset of GS:SFHS was followed-up with suicidal ideation elicited by self-report (*n* = 3342, suicidal ideation cases = 158) and coping styles measured by the Coping Inventory for Stressful Situations. The relationship of neuroticism to suicidal ideation, and the role of coping style, was then investigated using multivariable logistic regression.

**Results:**

Neuroticism was positively associated with hospital-associated self-harm in GS:SFHS (per EPQ-SF unit odds ratio 1.2 95% credible interval 1.1–1.2, *p*_FDR_ 0.0003) and UKB (per EPQ-SF unit odds ratio 1.1 95% confidence interval 1.1–1.2, *p*_FDR_ 9.8 × 10^−17^). Neuroticism, and the neuroticism-correlated coping style, emotion-oriented coping (EoC), were also associated with suicidal ideation in multivariable models.

**Conclusions:**

Neuroticism is an independent predictor of hospital-treated self-harm risk. Neuroticism and emotion-orientated coping styles are also predictive of suicidal ideation.

**Electronic supplementary material:**

The online version of this article (10.1007/s00127-019-01725-7) contains supplementary material, which is available to authorized users.

## Introduction

Suicide is a major global health challenge and is the leading cause of death among young people aged 20–34 years in the UK [69]. A variety of sociodemographic, biological and psychological risk factors have been proposed for completed suicide (for review, see [89]). Among the most predictive, and potentially amenable to clinical intervention, are (1) history of self-harm, which is associated with 37.2 times increased risk of completed suicide within the first year following an act of self-harm [70], and (2) suicidal ideation, which in a recent meta-analysis is associated with increased risk ratios for competed suicide of 2.35–8.00 [48].

Self-harm is a common and debilitating behaviour characterised by self-injury or self-poisoning, irrespective of the apparent purpose of the act [63]. Estimated lifetime prevalence of self-harm is 1–6%, with the UK reportedly having the highest self-harm rate in Europe [46]. Incidence is estimated at 400/100,000 population per year [90]. However, many people who self-harm do not attend clinical services, and thus true prevalence may be considerably greater [43].

Self-harm is aetiologically associated with childhood maltreatment [32, 84] and physical illness [23]. In addition, a number of demographic factors are predictive of self-harm, including being female [76]; young adulthood [76]; being unmarried [76]; or separated/divorced [72]; being socioeconomically disadvantaged [88]; unemployed [74]; or low educational attainment [75].

Psychiatric illness also has well-known associations with self-harm [79]. One systematic review of non-fatal self-injury presenting to hospital reported a pooled prevalence for psychiatric disorder of 83.9%, with mood disorders the most common category (58.5%) [44]. The association between depressive disorder and self-harm has been found in numerous other studies [6, 16].

Self-harm is performed with a variety of motivations, including attempted suicide, self-mutilation, seeking psychological relief, and the communication of distress. Often, there is not a single readily definable motivation, but multiple factors occurring simultaneously [20]. In the majority of cases, the intention is not to die [79]. Given the difficulties encountered clinically in ascertaining intent and motivation, it has been argued that the terms ‘deliberate self-harm’, ‘self-harm’, ‘attempted suicide’ and ‘suicidality’ are imprecise for research purposes [67]. Recently, the Fifth Edition of the Diagnostic and Statistical Manual for Mental Disorders [2] has proposed a distinction between ‘nonsuicidal self-injury’ (NSSI) and ‘suicidal behaviour disorder’ as ‘Conditions For Further Study’. However, it remains controversial whether such discrete categorizations can be confidently made in clinical practise, or demonstrate differentiable suicidal outcomes, given the biases inherent in self-report, and the close association of NSSI with suicidal behaviour [19, 52]. Broadly defined ‘self-harm’, therefore, retains an important clinical outcome in current suicidology literature [42, 52].

Another approach to subcategorising self-harm is on the basis of whether it has received hospital treatment. Hospital-treated self-harm is recognised as an important intervention point in suicide prevention [12]. Approximately one-seventh to one-fifth of those with hospital-treated self-harm will repeat their self-harm within 1 year [71]. Self-harm that requires medical attention significantly increases the future risk of suicide [19], particularly if admission to hospital is required [37]. Within the UK, up to one-fifth of those who die by suicide have attended hospital for self-harm in the preceding year [34].

Suicidal ideation, additionally, is an important antecedent to progression to significant self-harm and suicide attempts [32, 56]. Individuals who express suicidal ideation have significantly greater 12-month prevalence of self-harm and completed suicide, especially if there is associated planning [89]. Nevertheless, the relationship between self-harm and suicidal ideation is complex, with suicidal ideation having reportedly more than three times greater prevalence than suicide attempts [68].

Both self-harm and suicidal ideation are associated with personality, including personality disorders [41] and normally distributed personality traits. In particular, neuroticism is associated with suicidal ideation [22, 75], suicide attempts [73, 78], and suicide [24, 87]. A systematic review of personality traits and suicidality [9] found that neuroticism (and hopelessness) was the most predictive traits in risk screening. Neuroticism is a partially heritable personality trait which incorporates negative affectivity [29, 62] and increased sensitivity to stress (for review see [57]). An important aspect of neuroticism is that individual differences in the trait are moderately to highly stable over many years [17, 35] and thus might be useful as a patient level predictor for future self-harm risk. However, the link between neuroticism and self-harm is not wholly consistent and one large study did not find an association between neuroticism and lifetime history of prior suicide attempts [22].

Neuroticism is also highly correlated with affective disorder and both conditions show evidence of substantially overlapping genetic architecture [49, 54, 64]. There is uncertainty about whether neuroticism is a significant predictor of self-harm irrespective of depressive disorder history [31, 75] or whether it is insignificant when comorbid depression is controlled for [4, 8]. A recent study [75] in Chinese females concluded that neuroticism was significantly associated with suicide attempts even after controlling for comorbid depression and also stressful life events. Stressful life events are an additional posited factor in suicidal behaviour and it is hypothesised that neuroticism may serve to increase negative perceptions of these events [53, 73].

While considerable work has been undertaken at elucidating risk factors for self-harm and suicidal ideation, less is known about protective factors, which are not merely the absence of risk [79]. One component of managing adversity is coping styles, the behavioural and cognitive strategies adopted in response to stressful life events. These are not only situational, but may be environmentally and genetically conditioned [33]. They are of particular interest because they are potentially modifiable and might be impacted by treatment [14, 25].

Coping strategies are elicited by questionnaires like the Coping Inventory for Stressful Situations [27] which yields three main groups of coping strategies. The first is a “task-“or problem-oriented coping style (ToC), which is characterised by purposeful efforts aimed at problem solving. “Avoidance-orientated” (AoC) coping, in contrast, is defined by behaviours aimed at avoiding difficult circumstances [21]. Finally, “emotion-orientated” coping (EoC) is characterised by attempts to regulate difficult emotions as a means of coping. While ToC is generally seen as positively related to health and psychological adaptation, AoC and EoC are generally seen as less psychologically adaptive, and have been associated with negative mental health outcomes [45]. Task-oriented coping is thought to be negatively correlated with neuroticism [18] while emotion-oriented coping is positively correlated [28]. Moreover, emotion- and avoidance-oriented coping are thought to be associated with greater risk of suicidal ideation, while task-oriented coping is associated with lower risk [14].

In the first part of study, we aimed to investigate the relationship between neuroticism and hospital-treated self-harm. We employed two large UK population-based cohorts with neuroticism quantified by the same Eysenck Personality Questionnaire-Revised Short Form (EPQ-SF) scale. In one cohort, Generation Scotland (GS:SFHS), we used record-linkage to administrative health data to identify individuals with previous hospital-treated self-harm (generally defined and including all types of intentional self-injury requiring admission to medical or psychiatric hospital, *N* = 15,798; self-harm cases = 339). In the second cohort, UK Biobank (UKB), we used self-reported intentional self-harm (whether or not with intention to end life) requiring hospital treatment (including emergency department) and/or review by psychiatric services (*N* = 35,227; self-harm cases = 772). We hypothesised that neuroticism would be positively associated with self-harm, even after adjustment for depressive disorder and other significant sociodemographic factors.

In the second part of the study, we employed a follow-up sample of GS:SFHS with contemporaneous self-reported measures of suicidal ideation (*n* = 3356, suicidal ideation cases = 161). This follow-up group also had self-reported questionnaire data on significant life events and coping styles in response to stress. We hypothesised that neuroticism would also be independently predictive of suicidal ideation in this group, when adjusted for depressive disorder, significant life events and other significant demographic factors. We also aimed to ascertain the relationships on suicidal ideation of coping styles, particularly those correlated with neuroticism.

## Methods

### Cohorts

Generation Scotland:Scottish Family Health Study (GS:SFHS) is a population- and family based epidemiological adult (age 18+) cohort recruited February 2006–March 2011, which has been described elsewhere [80, 81]. GS:SFHS had a higher proportion of females (59%) and was of older age (mean 49 males, 49 females) compared to the Scottish population (mean 37 males, 39 females, 2001 census) [80]. GS:SFHS participants were typically healthier and more affluent that the general Scottish population, nevertheless 32.9% of individuals lived in areas with worse than average socioeconomic deprivation [80]. 99% of the study group was of white ethnicity (Scottish population 98%). Sociodemographic information on age, sex, educational attainment and relationship status were collected by questionnaire on enrolment.

Neuroticism was measured using the Eysenck Personality Questionnaire-Revised Short Form (EPQ-SF) [30]. The neuroticism subsection of the EPQ-SF consists of 12 ‘Yes/No’ questions (e.g., ‘Are you a worrier?’). Scores range from 0 to 12, with higher scores indicating greater neuroticism. This scale has been concurrently validated with other quantitative measures of neuroticism [39] and has high-reported reliability (*α*-coefficients 0.85–0.88) [30].

Trained researchers elicited lifetime history of major depressive disorder (MDD) using the screening questions from the Structured Clinical Interview for DSM-IV Disorders [80] and, if either screening question was positive, going on to administer the mood sections of the SCID. The screening questions were: “Have you ever seen anyone for emotional or psychiatric problems?” and “Was there ever a time when you, or someone else, thought you should see someone because of the way you were feeling or acting?”. A diagnosis of MDD was made according to DSM-IV criteria and all interviews were conducted by a trained researcher (2011 cases identified, 12.7% of cohort). Individuals with a history of bipolar disorder were excluded.

Cognitive testing included the digit symbol substitution test from the Wechsler Adult Intelligence Scale III [91], logical memory from the Wechsler Memory Scale III [92] and verbal fluency [58]. From these tests, a measure of cognitive function (g) was derived as the first unrotated principal component, explaining 44% of the variance in scores [59]. Socioeconomic deprivation was determined using the Scottish Index of Multiple Deprivation 2009 (SIMD) [77]. This measure employs 6976 geographical area-based data-zones across Scotland which are then ranked in order of deprivation, ascertained through weighted scores in seven domains including employment, education, health, housing and crime, with data-zone 1 the most deprived and 6976 the least deprived.

### Identification of self-harm

All Scottish citizens registered with a general practitioner are assigned a unique identifier, the Community Health Index (CHI). This was used to deterministically record-link GS:SFHS participants to the Scottish Morbidity Records to obtain information about hospital admissions (SMR01) and psychiatric hospital admissions (SMR04) associated with self-harm. Written informed consent was obtained from 98% of GS:SFHS and only those who consented were linked. Self-harm cases were identified by matching to admissions codes with E950–E959 (ICD-9) or X60–X84, Z915, E98 and Y1–Y3 (ICD-10) [5]. Scottish NHS data on mortality was also linked, to exclude any GS:SFHS participants who died during follow-up.

### Recontact group and identification of suicidal ideation in GS:SFHS

In 2014, GS:SFHS participants were recontacted for a follow-up assessment of mental health [66]. Suicidal Ideation was elicited using two questions from the General Health Questionnaire-28 [38]. Participants were asked *“*During the past few weeks…Have you thought of the possibility you might make away with yourself?” and “Have you found the idea of taking your own life kept coming into your mind?*”*. Participants who answered ‘Definitely have’ or ‘Has crossed my mind’ to either question were defined as suicidal ideation cases [*n* = 3503, cases = 158 (4.7%)].

Stressful life events were ascertained using the List of Threatening Experiences (LTE), whereby respondents self-reported their experiences from a list of 12 common threatening life events, occurring in the preceding 6 months [10, 11]. Examples of LTE include “Serious injury or assault to yourself”, “Made redundant or sacked from job” and “marital difficulties or break off of a steady relationship” (for full list see Supplementary Materials). For each event endorsed, contextual threat was rated on a scale from 3 (“very bad”) to 1 (“not too bad”). The LTE has demonstrated high test–retest reliability and good agreement with informant information (Cohen’s *κ* 0.63–0.90) [11].

Coping styles were elicited using the Coping Inventory for Stressful Situations (CISS) [28]; Cosway, [21], a 48 item self-report questionnaire enabling responders to rate on a 5-point scale their engagement in coping styles in response to stress, including task-, avoidance- and emotion-oriented coping. The CISS shows robust validity and reliability (alpha reliability coefficients (Cronbach’s alpha) of 0.82–0.90 for the main factors) [21]. History of MDD was re-ascertained using the Composite International Diagnostic Interview-Short Form (CIDI-SF) self-report questionnaire [55], with 605 cases identified (18.1% of sample). Bipolar disorder cases were excluded. Unlike the main GS:SFHS cohort, only one member from each family was analysed (i.e., unrelated sample).

### UKB

UK Biobank is a population-based cohort of adults aged 40–69 years recruited across the UK from 2006 to 2010, which has been described elsewhere [85]. During baseline assessment [82] participants provided sociodemographic information via a touch-screen questionnaire, including educational attainment and whether they lived as a singleton or couple. This study included a subset of 35,227 (7.0%) of UKB with complete case information for the variables of interest. Individuals in UKB who were also present in GS:SFHS (*n* = 201) were excluded.

Self-harm was ascertained through the touch-screen questionnaire. Participants were asked “Have you deliberately harmed yourself, whether or not you meant to end your life?”. A follow-up question enquired “Following any time when you took an overdose or deliberately tried to harm yourself did you (tick all that apply)”. Participants who ticked “see anyone from psychiatric or mental health services, including liaison services” and/or “need hospital treatment (e.g., A&E)” were included as cases in this study (772 cases, 2.2% of sample). The other answers, which were not included as cases, were “use a helpline”, “see own GP”, “receive help from friends/family” and “prefer not to answer”.

Neuroticism was assessed using the EPQ-SF [30], administered via the touch-screen questionnaire. Lifetime history of depression was ascertained by touch-screen questionnaire relating to lifetime experience of depressive symptoms and contact with mental health services [82].

Cognitive testing was administered via three touch-screen tests: (1) a symbol matching task over 12 trials (reaction time); (2) 13 logic/reasoning questions over 2 min (verbal-numerical reasoning); (3) card pair matching task (visuo-spatial memory). From these tests a single measure of cognitive ability (g) was extracted as the first unrotated principal component, explaining 42% of the variance.

Socioeconomic deprivation was measured via the Townsend Deprivation Index, a census-based measure incorporating unemployment, non-home ownership, household overcrowding and non-car ownership [50]. Each small postcode-based geographical area is assigned a Townsend Score, with zero indicating mean deprivation, negative scores indicating relative affluence, and positive scores indicating relative deprivation.

### Statistical analysis

All analyses were carried out using R version 3.2.3. Complete case analysis was employed in both cohorts (see Supplementary Table S4 for analysis of complete case versus whole-cohort variables). Generalised linear models with logit-link function (logistic regression) were used to identify predictors of self-harm in UK Biobank. In the GS:SFHS self-harm study, additional adjustment for inter-relatedness of the family-based cohort was performed using a Bayesian mixed model approach, with pedigree fitted as a random effect, using an inverse pedigree matrix within the R package MCMCglmm. This implements a Markov Chain Monto Carlo estimator, with a “threshold” family probit link function which produces similar results to a logit function, optimised to pedigree based mixed effects models. In the GS:SFHS and UKB multivariable analyses of hospital-treated self-harm, predictor variables are reported unstandardized.

In the GS:SFHS suicidal ideation follow-up study, an unrelated sample was used and multivariable logistic regression was employed. In this analysis, continuous variables were scaled to have a mean of zero and standard deviation of one, to facilitate interpretation of the CISS and LTE predictor variables. During fitting of models, interaction terms for neuroticism and depression, and neuroticism and coping styles, were tested to investigate potential moderation on neuroticism.

Coefficients were expressed as odds ratios with 95% credible intervals and 95% confidence intervals as applicable. *p* values were reported after False Discovery Rate adjustment [7]. Group differences between numeric variables were ascertained using Cohen’s *t* test and Cohen’s *d* measure of effect size, and differences between proportions were assessed using *z* test and Cohen’s *h*. For all experiments, we have reported all measures, conditions, data exclusions and the determination of sample sizes and further information is available in Supplementary Table S4.

## Results

### GS:SFHS

As presented in Table 1, there were 339 (2.1%) GS:SFHS individuals identified with previous self-harm requiring hospital admission. Self-harm cases were slightly younger (mean age 44.7 versus 47.1, *p* = 0.0005, Cohen’s *d *= 0.16), predominantly female (66.7% versus 58.4%, *p* 0.002, Cohen’s *h *= 0.17), with lower mean cognitive ability scores, greater prevalence of depression history (47.5% versus 12%, *p* < 2.2 × 10^−16^, *h *= 0.81) and with higher mean neuroticism (mean 6.4 versus 3.7, *p* < 2.2 × 10^−16^, *d *= 0.89). Self-harm cases were more likely to be from more deprived areas as measured by SIMD (mean 1964 versus 1823, *p* < 2.2 × 10^−16^, *d *= 0.58). The proportion of graduates was lower in self-harm cases (17.1% versus 33.9%, *p* < 10.0 × 10^−11^, *h *= 0.39). A greater proportion of self-harm cases reporting being single (51.9% versus 31.7%, *p* < 3.6 × 10^−15^, *h *= 0.31).

**Table 1:** Socio-demographic, clinical and cognitive characteristics of GS:SFHS (*N* = 15798) and UK Biobank (*N* = 35227) cohorts used in this study

	GS:SFHS (*N* = 15798)		*p* value (effect size)	UKB (*N* = 35227)		*p* value (effect size)
	Self-harm (%/s.d.)	Controls (%/s.d.)		Self-harm (%/s.d.)	Controls (%/SFD)	
Total	339 (2.1)	15,459		772 (2.2)	34,455	
Female	226 (66.7)	90,28 (58.4)	0.002 (0.17)	544 (70.5)	18,591 (54.0)	< 2.2 × 10^−16^ (0.34)
Age	44.7 (12.3)	47.1 (15.0)	0.0005 (0.16)	53.3 (7.6)	56.6 (7.7)	< 2.2 × 10^−16^ (0.43)
Age categories: 18–24	18 (5.3)	1488 (9.6)				
25–34	57 (16.8)	2058 (13.3)				
35–44 GS:SFHS/40–44 UKB	94 (27.7)	2871 (18.6)		113 (14.6)	3215 (9.3)	
45–54	87 (25.7)	3361 (21.7)		311 (40.3)	9588 (27.8)	
55–64	71 (20.9)	4113 (26.6)		300 (38.9)	16,025 (46.5)	
65–74	10 (2.9)	1245 (8.1)		48 (6.2)	5627 (16.3)	
75+	2 (0.6)	323 (2.1)				
History of depression	161 (47.5)	1850 (12.0)	< 2.2 × 10^−16^ (0.81)	699 (90.5)	11,474 (33.3)	< 2.2 × 10^−16^ (1.3)
EPQ neuroticism (mean)	6.4 (3.5)	3.7 (3.1)	< 2.2 × 10^−16^ (0.89)	5.6 (3.1)	3.3 (2.8)	< 2.2 × 10^−16^ (0.83)
Cognitive ability scores (mean):						
Verbal declarative	15.5 (4.4)	16.3 (3.9)	0.003 (0.19)			
Vocabulary	28.4 (4.8)	30.3 (4.7)	< 5.4 × 10^−12^ (0.40)			
Processing speed	67.3 (16.9)	73.1 (16.9)	< 1.0 × 10^−9^ (0.34)			
Executive function	23.8 (8.2)	25.9 (8.1)	< 4.4 × 10^−6^ (0.26)			
Visual memory				1.4 (0.6)	1.4 (0.6)	0.20
Verbal-numerical reasoning				6.8 (2.1)	6.7 (2.1)	0.25
Reaction time				6.3 (0.2)	6.3 (0.2)	0.10
SIMD rank (mean, most deprived rank 1, least deprived rank 6976)	2918 (1964)	3993 (1823)	< 2.2 × 10^−16^ (0.58)			
Townsend score (mean)				− 0.5 (3.1)	− 1.7 (2.6)	< 2.2 × 10^−16^ (0.44)
Education: No qualification or other	83 (24.5)	1897 (12.3)	< 1.84 × 10^−11^ (0.32)	34 (4.4)	2082 (6.0)	0.06
O-levels/GCSEs	52 (15.3)	1882 (12.2)		155 (20.1)	6907 (20.1)	
CSE or equivalent				37 (4.8)	1330 (3.9)	
A-levels or equivalent	29 (8.6)	1808 (11.7)		116 (15.0)	4712 (13.7)	
NVQ or equivalent	117 (34.5)	4636 (30.0)		39 (5.1)	1773 (5.2)	
Other professional				33 (4.3)	1802 (5.2)	
College or university degree	58 (17.1)	5236 (33.9)	< 10.0 × 10^−11^ (0.39)	358 (46.4)	15,849 (46.0)	0.83
Living as single	176 (51.9)	4906 (31.7)	< 3.6 × 10^−15^ (0.31)	306 (39.6)	7930 (23.0)	< 2.2 × 10^−16^ (0.36)

Percentages are shown in brackets for categorical variables and standard deviations for continuous variables. Probability (*p*) values are derived from Cohen’s *t* tests for continuous variables and *z* tests for proportions. Effect sizes are derived from Cohen’s *d* for numeric variables and Cohen’s *h* for categorical variables. Townsend scores are standardised—positive values of the index indicate areas of high material deprivation, negative values indicate relative affluences, and score 0 indicates mean values

*GS:SFHS* Generation Scotland, *UKB* UK Biobank, *SIMD* Scottish Index of Multiple Deprivation, *s.d.* standard deviation, *O-levels/GCSEs* ordinary level (Year 11)school certificate, *CSE* Certificate of Secondary Education(Year 11), *A-levels* Advanced level (Year 13)school certificate

**Fig. 1:**
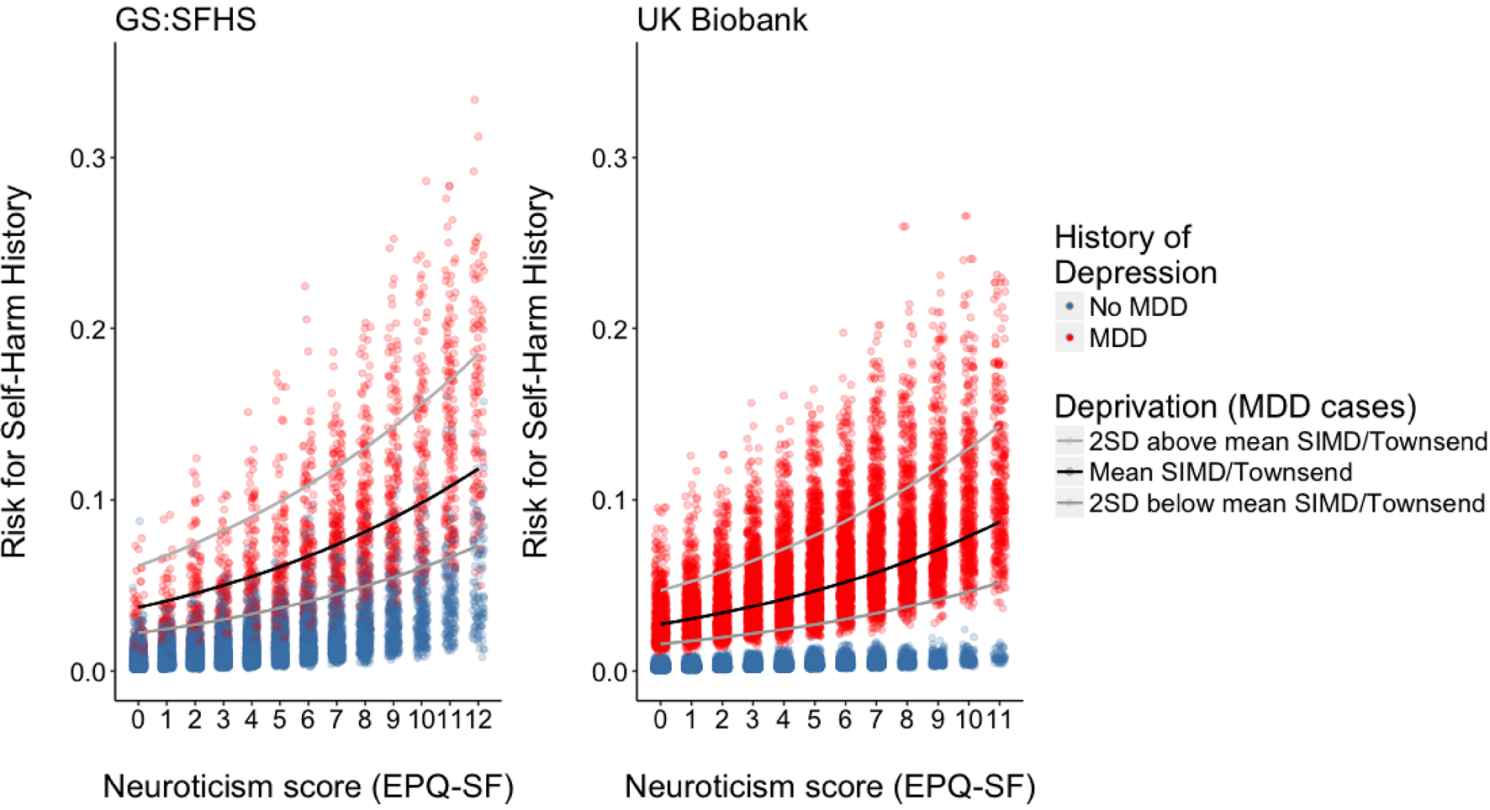
Predicted risk of self-harm from the multivariable models in GS:SFHS and UKB for different EPQ-SF neuroticism scores

The most predictive factor for previous self-harm (Table 2) was history of major depressive disorder [OR 5.6 95% credible interval (CI) 3.5–8.9, *p*_FDR_ 0.0004]. Neuroticism was positively associated with self-harm risk by an odds ratio of 1.2 (95% CI 1.1–1.2, *p*_FDR_ = 0.0003) per EPQ-SF unit. No significant interaction terms were found during model fitting. The significant effects of neuroticism were found in both male-only and female-only combined models (see Supplementary Table S1). Figure 1 displays the increased risk of self-harm per unit of EPQ-SF neuroticism score predicted by our model for both cohorts.

**Table 2:** Multivariable analysis of predictors of history of self-harm involving hospital/psychiatric treatment in GS:SFHS and UKB [comparison made to any reported history of self-harm in UKB (*)]

	GS:SFHS	Self-harm with hospital attendance	UKB	Self-harm with hospital attendance	UKB(*)	Any reported self-harm*
Cases (%)	339 (2.1%)		772 (2.2%)		1578 (4.4%)	

	Odds ratios	*p*_FDR_	Odds ratios	*p*_FDR_	Odds ratios	*p*_FDR_
Gender						
Male	Ref		Ref		Ref	
Female	1.1 (0.8–1.4)	0.67	1.3 (1.1–1.5)	0.005 (*)	1.3 (1.1–1.4)	0.0001 (***)
Age						
18–24	0.5 (0.3–1.0)	0.07 (.)	–	–	–	–
25–34	2.0 (1.2–3.3)	0.01 (*)	–	–	–	–
35–44	2.2 (1.4–3.5)	0.0005 (**)	1.4 (1.1–1.7)	0.03 (*)	2.1 (1.8–2.4)	2.2 × 10^−19^ (***)
45–54	1.6 (1.1–2.5)	0.03 (*)	1.4 (1.2–1.7)	0.0003 (**)	1.7 (1.5–1.9)	1.9 × 10^−15^ (***)
55–64	Ref		Ref		Ref	
64–74	0.4 (0.2–0.8)	0.02 (*)	0.6 (0.5–0.9)	0.01 (*)	0.7 (0.5–0.8)	0.0009 (**)
75+	0.2 (0.04–0.97)	0.04 (*)	–	–	–	–
No history of depression	Ref		Ref		Ref	
History of Depression	5.6 (3.5–8.9)	0.0004 (**)	12.7 (9.9–16.4)	5.4 × 10^−86^ (***)	6.4 (5.5–7.3)	1.0 × 10^−139^ (***)
EPQ Neuroticism	1.2 (1.1–1.2)	0.0003 (**)	1.1 (1.1–1.2)	9.8 × 10^−17^ (***)	1.1 (1.1–1.2)	3.4 × 10^−41^ (***)
Cognitive function (g)	0.8 (0.7–0.9)	0.0005 (**)	1.1 (1.0–1.2)	0.051 (.)	1.1 (1.0–1.1)	0.004 (*)
Education						
No qualification or other	2.2 (1.2–4.1)	0.02 (*)	1.0 (0.6–1.4)	0.96	0.9 (0.6–1.2)	0.47
O-levels	1.1 (0.7–2.1)	0.67	1.0 (0.8–1.3)	0.97	1.0 (0.8–1.2)	0.94
CSE or equivalent			1.0 (0.7–1.5)	0.98	0.9 (0.7–1.2)	0.56
A-levels or equivalent	Ref		Ref		Ref	
NVQ or equivalent	1.4 (0.8–2.4)	0.23	1.1 (0.8–1.6)	0.77	1.0 (0.7–1.3)	0.99
Other professional			0.9 (0.6–1.4)	0.96	1.0 (0.8–1.4)	0.94
College or university degree	0.7 (0.4–1.2)	0.20	1.0 (0.8–1.2)	0.96	1.1 (1.0–1.3)	0.20
SIMD quintile (increased score, less socioeconomically deprived)	0.8 (0.7–0.9)	0.0004 (**)				
Townsend score (increased score, more socioeconomically deprived)			1.1 (1.1–1.1)	9.0 × 10^−14^ (***)	1.1 (1.1–1.1)	7.1 × 10^−16^ (***)
Living as couple	Ref		Ref		Ref	
Living as single	2.0 (1.5–2.8)	0.0003 (**)	1.3 (1.1–1.5)	0.005 (*)	1.3 (1.1–1.4)	0.00006 (***)

95% credible (GS:SFHS) and confidence(UKB) intervals are shown in brackets for odds ratios. Significance indicators are *p < 0.05, **p<0.001, ***p<0.0001

*GS:SFHS* Generation Scotland cohort, *UKB* UK Biobank cohort, *pFDR p* value using False Discovery Rate method, *EPQ* Eysenck Personality Questionnaire, *SIMD* Scottish Index of Multiple Deprivation, *NVQ* National Vocational Qualification, *Ref* reference category, *O-levels/GCSEs* ordinary level (Year 11)school certificate, *CSE* Certificate of Secondary Education (Year 11), *A-levels* Advanced level (Year 13)school certificate

The age groups 25–34, 35–44, and 45–54 were positively associated with self-harm whereas age groups 64–74 and 75+ were negatively associated, compared to the reference category of 55–64. Gender did not show a significant association in the combined model. Having a higher SIMD score (less deprived) was associated with decreased risk of self-harm (per quintile unit OR 0.8 95% CI 0.7–0.9, *p*_FDR_ = 0.0004). Having no qualifications and being single increased risk. Cognitive ability showed an inverse association with self-harm (per unit OR 0.8; 95% CI 0.7–0.9, *p*_FDR_ = 0.0005).

### UKB

There were 772 (2.2%) individuals self-reporting self-harm requiring hospital or psychiatrist review in UKB (Table 1). Self-harm cases were slightly younger (UKB’s minimum age is 40), predominantly female (70.5% versus 54.0%, *p* < 2.2 × 10^−16^, *h *= 0.34), and with higher mean neuroticism (mean 5.6 versus 3.3, *p* < 2.2 × 10^−16^,*d *= 0.83) and higher prevalence of history of depression (90.5% versus 33.3%, *p* < 2.2 × 10^−18^, *h *= 1.3). Cognitive ability scores were not significantly different for any of the tests. Self-harm cases were more likely to be from deprived areas (more positive scores) as measured by the Townsend index (mean − 0.5 versus − 1.7, *p* < 2.2 × 10^−16^, *d* = 0.44). Educational attainment was not significantly different between the two groups (χ = 7.43, *p* value 0.28). The proportion of the self-harm group living as single was 39.6% versus 22.5% for those reporting no history of self-harm.

The most predictive factor in the multivariable logistic model was self-reported history of depression (Table 2, OR 12.7 95% confidence interval (CI) 9.9–16.4, *p*_FDR_ 5.4 × 10^−86^). The odds of self-harm were significantly positively associated with increasing neuroticism scores, OR 1.1 95% CI 1.1–1.2, *p*_FDR_ 9.8 × 10^−17^ per EPQ-SF unit. No significant interactions were found during model fitting. The significant effects of neuroticism were found in both the male-only and female-only models (Supplementary Table S1).

Being female was also associated with somewhat higher risk (OR 1.3 95% CI 1.1–1.5, *p*_FDR_ 0.005), as was being in the 35–44 and 45–54 age groups, whereas the 65–74 age group was protective. No educational factors were significant in the multivariable analysis. Being single and higher Townsend scores (more deprived) were associated with higher odds of self-harm. Cognitive ability (g) was not significantly associated (*p*_FDR_ = 0.051, OR 1.1, 95% CI 1.0–1.2).

In Table 2, comparison is also made to UKB participants who self-reported any self-harm, irrespective of whether hospital attention was sought (1578 cases, 4.4%). In this group self-harm was also positively associated with neuroticism scores (OR 1.1, 95% CI 1.1–1.2, *p*_FDR_ 3.4 × 10^−41^ per EPQ-SF unit). Positive association was also found for history of depression, being female, younger age group, increasing Townsend deprivation score, and being single. However, in this group increasing cognitive function score increased odds of self-harm (OR 1.1, 95% CI 1.0–1.1, *p*_FDR_ 0.004 per unit g).

### GS:SFHS suicidal ideation recontact study

In the GS:SFHS recontact study (*N* = 3342) there were 158 individuals with self-reported suicidal ideation(4.7%) (Table 3). Of these 21 (13.3%) had a record-linkage based history of self-harm compared to 1.9% in the control group. History of self-harm was the most predictive factor for suicidal ideation in the multivariable model (OR 3.5, 95% CI 1.9–6.2, *p*_FDR_ 7.5 × 10^−5^) followed by history of depression (OR 3.2, 2.3–4.7, *p*_FDR_ = 7.8 × 10^−10^). Scores in the List of Threatening Experiences increased odds of suicidal ideation (1.3, 1.2–1.5, *p*_FDR_ = 2.4 × 10^−5^ per standard deviation unit).

**Table 3:** Multivariable analysis of predictors of history of suicidal ideation in GS:SFHS Re-Contact Study (*N* = 3342)

	Odds ratios		Multivariable model including coping styles OR (95% CI, *p*_FDR_ value)
	Univariate model OR(95% CI, *p*_FDR_ value)	Multivariable model OR(95% CI, *p*_FDR_ value)	
Female gender	0.08 (0.5–1.0) *p* = 0.08	**0.5 (0.3–0.7)** ***p* = 1.4 × 10**^**−4**^	**0.4 (0.3–0.7)** ***p* = 1.0 × 10**^**−4**^
Age	**0.08 (0.7–1.0)** ***p* = 0.01**	0.9 (0.8–1.1) *p* = 0.21	1.0 (0.9–1.2) *p* = 0.85
History of depression (CIDI)	**4.7 (3.4–6.4)** ***p* = 2.3 × 10**^**−20**^	**3.2 (2.3–4.7)** ***p* = 7.8 × 10**^**−10**^	**2.3 (1.6–3.4)** ***p* = 5.8 × 10**^**−5**^
EPQ Neuroticism score*	**1.9 (1.7–2.2)** ***p* = 4.0 × 10**^**−20**^	**1.6 (1.3–1.8)** ***p* = 5.8 × 10**^**−8**^	1.1 (0.9–1.4) *p* = 0.44
Cognitive ability(g)*	**0.8 (0.7–0.9)** ***p* = 0.002**	0.9 (0.7–1.1) *p* = 0.21	0.9 (0.7–1.0) *p* = 0.15
Socioeconomic deprivation (SIMD) rank*	**0.7 (0.6–0.9)** ***p* = 1.2 × 10**^**−4**^	0.9 (0.8–1.1) *p* = 0.26	0.9 (0.8–1.1) *p* = 0.36
History of self-harm	**8.1 (4.76–13.5)** ***p* = 6.5 × 10**^**−15**^	**3.5 (1.9–6.2)** ***p* = 7.5 × 10**^**−5**^	**3.2 (1.7–5.8)** ***p* = 6.1 × 10**^**−4**^
List of threatening experiences total*	**1.4 (1.3–1.6)** ***p* = 2.0 × 10**^**−10**^	**1.3 (1.2–1.5)** ***p* = 2.4 × 10**^**−5**^	**1.3 (1.1–1.5)** ***p* = 4.2 × 10**^**−4**^
CISS emotion oriented coping*	**2.9 (2.4–3.5)** ***p* = 4.0 × 10**^**−33**^		**2.4 (1.9–3.0)** ***p* = 1.7 × 10**^**−12**^
CISS task-oriented coping*	**0.6 (0.5–0.7)** ***p* = 3.3 × 10**^**−13**^		**0.8 (0.6–0.9)** ***p* = 0.03**
CISS Avoidance-oriented coping*	0.9 (0.8–1.1) *p* = 0.33		0.8 (0.7–1.0) *p* = 0.15

95% confidence intervals are shown in brackets for odds ratios

Significant values are in bold (*p* ≤ 0.05)

For comparison of proportions/mean scores of independent variables between history of suicidal ideation cases and controls, see Table S3 in Supplementary Material

*OR* Odds Ratio, *95% CI* 95% confidence interval, *EPQ* Eysenck Personality Questionnaire-revised Short Form, *SIMD* Scottish Index of Multiple Deprivation, *CISS* Coping inventory for stressful situations, *CIDI* composite international diagnostic interview

*Continuous variables have been scaled to have a mean of zero and standard deviation of one

Neuroticism was positively associated with suicidal ideation in the multivariable model (OR 1.6, 1.3–1.8, *p*_FDR_ = 5.8 × 10^−8^ per standard deviation unit). However, this association attenuated to non-significant OR 1.1 (0.9–1.4, *p* = 0.44) when coping styles were added to the model (Table 3). In the full multivariable model including coping styles, EoC was positively associated with suicidal ideation (OR 2.4, 1.9–3.0, *p*_FDR_ = 1.7 × 10^−12^) and ToC was negatively associated (OR 0.8, 0.8–0.9, *p*_FDR_ = 0.03), while AoC was not significantly associated. The correlation matrix revealed that EoC and neuroticism were significantly correlated, *r* = 0.50 *p* < 2.2 × 10^−16^ and task-oriented coping were moderately negatively correlated [*r* = − 0.18 *p* < 2.2 × 10^−16^, Table S2 (Supplementary)]. In moderation analysis no significant (*p* ≤ 0.05) interaction terms were found for neuroticism*ToC, neuroticism*AoC or neuroticism*EoC on suicidal ideation, controlled for age, sex and depression status.

## Discussion

Here we report a significant independent association between neuroticism and history of self-harm requiring medical attention in two large population-based cohorts, using both self-reported and record-linkage derived measures of self-harm. This finding remained significant when controlling for history of depression, socioeconomic deprivation, educational attainment and relationship status.

In both UKB and GS:SFHS we found that history of depression was the predictor with largest effect size on hospital-treated self-harm risk. In our multivariable models, predicted self-harm risk (Fig. 1) was relatively low in UKB in non-depressed individuals even at higher neuroticism scores, whereas in GS:SFHS more neurotic non-depressed cases also had significant overall risk. This disparity may be explained by the use of self-reported depression in UKB, with broader inclusion criteria than GS:SFHS (which employed the objectively assessed SCID). Thus, 90.5% of self-harm cases reported history of depression in UKB, versus 47.5% in GS:SFHS (Table 1).

We found a significant protective relationship for higher cognitive scores against self-harm in GS:SFHS, but not in UKB. Previous studies have found that cognitive impairment is associated with suicide and self-harm [1, 5, 40, 51, 83]. However, other studies have found increased cognitive scores may increase self-harm risk [3, 13]. One explanation for the discrepancy in our results is that different measures of cognitive ability were used in the two cohorts (Table 1). Moreover, previous research on depression and cognitive ability in GS:SFHS and UKB [65] has been similarly inconclusive, with an association between g and depression being identified in GS:SFHS but not UKB. In terms of education attainment, we found fewer graduates and more individuals without qualifications in self-harm cases in GS:SFHS, but this difference was not significant in UKB. This might be accounted for in population sampling differences between GS:SFHS and UKB, with the latter having more graduates among controls also (Table 1).

We found socioeconomic deprivation was significantly associated with self-harm history in both cohorts, as was living as a singleton. Female gender was not predictive of self-harm in GS:SFHS but was significantly associated in UKB, albeit with modest effect size (Table 2). Previous multi-centre studies have shown female rates of self-harm to be significantly higher than male [76]. However, our GS:SFHS analysis was for hospital inpatient admitted self-harm and it may be that in this subgroup female gender is less predictive of risk, given that hospital-treated self-harm arguably lies on a spectrum between non-serious self-harm and suicide, the latter of which is four times more common in males [60].

In our follow-up analysis of suicidal ideation, we found an independent association between neuroticism and self-reported suicidal ideation, which remained significant when controlled for history of depression, socioeconomic deprivation and significant life events. When coping styles were added to the model, the association with suicidal ideation was no longer significant, implying that neuroticism’s effect is not independent of coping style. We showed that emotion-orientated coping is highly positively correlated with neuroticism (*r* = 0.50) and task-orientated coping negatively correlated (*r* = − 0.18). In addition, we found that emotion-oriented coping was positively associated with suicidal ideation whereas task-oriented coping was negatively associated. This relationship was also found in a study of suicidal ideation in middle-aged workers in Japan, albeit without employing a validated coping style instrument [86]. A further study found emotion-focused coping, but not problem-focused coping, was associated with suicidal ideation in adolescents [47]. “Active” (task-oriented) coping and positive reinterpretation were also associated with lower suicidality, adjusted for depression, in a study of 500 college students [14].

### Study strengths and limitations

This study had a number of strengths for establishing the association of neuroticism to hospital-treated self-harm. We have employed two large, population-based cohorts which both have phenotypic information for major covariates of self-harm, allowing comparison between the groups while both using the same EPQ-SF measure of neuroticism. By utilising self-report in one cohort, and health data record-linkage in the other, our study design obviates some of the biases which can arise from utilising either method alone. GS:SFHS encompasses the range of adult age groups, and UKB focuses on middle-age to older adults, thus our findings are a significant contribution to self-harm research where many of the available studies are for teenagers or young adults. By extending our analysis to suicidal ideation, we were also able to demonstrate an association with neuroticism and correlated coping styles (emotion- and task-oriented coping), the latter of which are potentially modifiable by clinical intervention.

There are also some important limitations to our work. The cohorts we use are population-based, but are not fully representative, as UKB includes adults of ages 40–69 and GS:SFHS has an older mean age that the Scottish population. Additionally, the use of GP registration as an inclusion criteria for our GS:SFHS study (by enabling record-linkage via CHI number) leads to potential selection bias in our identification of self-harming individuals, although in the UK 96% of individuals are registered with a GP [80] indicating that such biases are likely to be small. The prevalence of self-harm we record should thus be used with caution and should not be taken as a reliable population estimate. Nevertheless, it is sobering that prevalence of hospital-treated self-harm was relatively high (2.1% for GS:SFHS and 2.2% for UKB). Since self-harm is more common in younger people, the true population prevalence is likely to be greater still. We have also adopted a cross-sectional design and thus causality between factors such as neuroticism and self-harm; and neuroticism, coping style and suicidal ideation; is suggested rather than conclusively demonstrated by our models.

The type of self-harm we have studied is self-harm involving hospital care. We used a general definition of self-harm as the data available to us did not allow distinction between nonsuicidal self-injury and suicide attempts, as this information is not available in the routinely collected administrative hospital data linked to in GS:SFHS (and was not part of the self-report question in UKB). This could limit the transferability of our results to other studies, although as discussed, the extent to which such distinctions of suicidal intent can be accurately made in practise is controversial.

In GS:SFHS we defined self-harm cases via admission to medical or psychiatric hospital, as ascertained by record-linkage. We therefore have not included a number of self-harm cases that were managed in the Emergency Department, where available data is incomplete [61]. This represents approximately 50% of self-harm cases presenting to hospital, although there are wide variations between hospitals [20]. A recent study has found that routine hospital data underestimates rates of self-harm by approximately 60% compared to combined survey-hospital database methods [15], as—for example—self-harm which is assessed in the Emergency Department, but which does not lead to hospital admission, may not be included. However, hospital admission self-harm is itself an important variable, as cases that are admitted are likely to be more serious and can therefore be expected to be of greater risk of further self-harm and completed suicide [37]. The UKB self-report variable was for self-harm requiring any hospital or psychiatric management (including Emergency Department) and therefore, while highly correlated with the GS:SFHS variable, was more general in its scope. The overall prevalence of self-harm in GS:SFHS and UKB was similar (2.1% and 2.2%, respectively). This might seem surprising as one might expect the more general self-harm definition in UKB to return a higher prevalence. This could be explained by the fact that the UKB cohort had no individuals younger than 40 and this has decreased the overall self-harm prevalence, since younger age groups are at relatively higher risk.

We employed a complete case design in our multivariable analyses in GS:SFHS and UKB. Potentially, this could have biased our results compared to the whole samples, although comparison (Supplementary Table S4) indicated that there were no significant and large-effect differences in major variables studied through the complete case approach. Nevertheless, this method could have introduced biases in ways we did not measure.

In summary, our findings must be seen in the context of self-harm with a high propensity to cause physical harm warranting medical attention. However, the UKB cohort did include a variable for any self-harm regardless of hospital attendance and we also included this multivariable analysis (Table 2). Neuroticism was found to be associated in this group also, with similar effect size (OR 1.1, 95 CI 1.1–1.2, *p* 3.4 × 10^−41^ per EPQ-SF unit).

With regard to our analysis of suicidal ideation and coping-style, neuroticism as a trait was measured during GS:SFHS enrolment, which was some years before the recontact when coping style and suicidal ideation were measured. However, as discussed, neuroticism is considered to be a relatively stable trait and would not be expected to change significantly over this time period. We also controlled neuroticism by age at enrolment rather than age at recontact within the models. Another important consideration is the extent to which neuroticism and emotion-oriented coping are separate constructs or both emanant from innate responses to stress. While we found the correlation of neuroticism and EoC to be significant (0.5), it was evidently not complete. There is also evidence that coping style is amenable to clinical treatment in prevention of suicide [36], whereas personality traits are understood as more therapeutically static.

## Conclusion and implications for practice

We have found that a questionnaire which is relatively quick to administer in a clinical setting, the EPQ-SF, is significantly independently predictive of self-harm and suicidal ideation when adjusted for multiple other significant factors, including history of depression. Neuroticism is, therefore, an important factor which should be included in future studies of self-harm and suicidality risk.

Our research also implies a potential role for cognitive-behavioural therapies focused on decreasing emotion-oriented coping and increasing adaptive task-oriented coping in individuals with suicidal ideation. There is current limited research in this area, although previous studies are encouraging [25, 26]. The coping styles questionnaires are also relatively straightforward to administer clinically and our study suggests that greater attention to reducing emotion-orientated coping, and reinforcing task-oriented coping strategies, in individuals presenting with suicidal ideation is likely to have a beneficial effect in protecting against self-harm.

We also demonstrate the utility of record-linkage to health data for examining research variables such as self-harm, where there may be an unwillingness to self-report caseness, but a willingness to provide consent for anonymised data linkage. Such record-linked cohort studies provide an important new avenue for future research on self-harm and psychiatric illness.

## Electronic supplementary material

Below is the link to the electronic supplementary material.

Supplementary material 1 (DOCX 41 kb)

## References

[1] Alati R, Gunnell D, Najman J et al (2009) Is IQ in childhood associated with suicidal thoughts and attempts? Findings from the Mater University Study of Pregnancy and its outcomes. Suicide Life Threat Behav 39(3):282–293 19606920 10.1521/suli.2009.39.3.282

[2] American Psychiatric Association (2013) Diagnostic and statistical manual of mental disorders, 5th edn. American Psychiatric Association, Washington DC

[3] Apter A, Bleich A, King RA et al (1993) Death without warning? A clinical postmortem study of suicide in 43 Israeli adolescent males. Arch Gen Psychiatry 50(2):138–142 8427554 10.1001/archpsyc.1993.01820140064007

[4] Batterham PJ, Christensen H (2012) Longitudinal risk profiling for suicidal thoughts and behaviours in a community cohort using decision trees. J Affect Disord 142(1–3):306–314 22840465 10.1016/j.jad.2012.05.021

[5] Batty GD, Whitley E, Deary IJ et al (2010) Psychosis alters association between IQ and future risk of attempted suicide: cohort study of 1,109,475 Swedish men. BMJ 340:c2506 20522657 10.1136/bmj.c2506 PMC2881197

[6] Beautrais AL (2000) Risk factors for suicide and attempted suicide among young people. Aust N Z J Psychiatry 34(3):420–436 10881966 10.1080/j.1440-1614.2000.00691.x

[7] Benjamini Y, Hochberg Y (1995) Controlling the false discovery rate: a practical and powerful approach to multiple testing. J R Stat Soc Ser B (Methodological) 57(1):289–300

[8] Bi B, Xiao X, Zhang H et al (2012) A comparison of the clinical characteristics of women with recurrent major depression with and without suicidal symptomatology. Psychol Med 42(12):2591–2598 22716960 10.1017/S003329171200058X PMC3488812

[9] Brezo J, Paris J, Turecki G (2006) Personality traits as correlates of suicidal ideation, suicide attempts, and suicide completions: a systematic review. Acta Psychiatr Scand 113(3):180–206 16466403 10.1111/j.1600-0447.2005.00702.x

[10] Brugha T, Bebbington P, Tennant C et al (1985) The list of threatening experiences: a subset of 12 life event categories with considerable long-term contextual threat. Psychol Med 15(1):189–194 3991833 10.1017/s003329170002105x

[11] Brugha TS, Cragg D (1990) The list of threatening experiences: the reliability and validity of a brief life events questionnaire. Acta Psychiatr Scand 82(1):77–81 2399824 10.1111/j.1600-0447.1990.tb01360.x

[12] Carroll R, Metcalfe C, Gunnell D (2014) Hospital presenting self-harm and risk of fatal and non-fatal repetition: systematic review and meta-analysis. PLoS ONE 9(2):e89944 24587141 10.1371/journal.pone.0089944 PMC3938547

[13] Chang SS, Chen YY, Heron J et al (2014) IQ and adolescent self-harm behaviours in the ALSPAC birth cohort. J Affect Disord 152–154:175–182 24080206 10.1016/j.jad.2013.09.005

[14] Chou W-J, Ko C-H, Hsiao RC, Cheng C-P, Yen C-F (2017) Association of stress coping strategies with suicidality in young adults: the mediation effects of depression. Anxiety Hostility Neuropsychiatry 7(6):974–982

[15] Clements C, Turnbull P, Hawton K et al (2016) Rates of self-harm presenting to general hospitals: a comparison of data from the multicentre study of self-harm in England and hospital episode statistics. BMJ Open 6(2):e009749 26883238 10.1136/bmjopen-2015-009749 PMC4762081

[16] Colman I, Newman SC, Schopflocher D et al (2004) A multivariate study of predictors of repeat parasuicide. Acta Psychiatr Scand 109(4):306–312 15008805 10.1111/j.1600-0447.2003.00282.x

[17] Conley JJ (1985) Longitudinal stability of personality traits: a multitrait-multimethod-multioccasion analysis. J Pers Soc Psychol 49(5):1266–1282 4078676 10.1037//0022-3514.49.5.1266

[18] Connor-Smith JK, Flachsbart C (2007) Relations between personality and coping: a meta-analysis. J Pers Soc Psychol 93(6):1080–1107 18072856 10.1037/0022-3514.93.6.1080

[19] Cooper J, Kapur N, Webb R et al (2005) Suicide after deliberate self-harm: a 4-year cohort study. Am J Psychiatry 162(2):297–303 15677594 10.1176/appi.ajp.162.2.297

[20] Cooper J, Steeg S, Bennewith O et al (2013) Are hospital services for self-harm getting better? An observational study examining management, service provision and temporal trends in England. BMJ Open 3(11):e003444 24253029 10.1136/bmjopen-2013-003444 PMC3840333

[21] Cosway R, Endler NS, Sadler AJ, Deary IJ (2000) The coping inventory for stressful situations: factorial structure and associations with personality traits and psychological health. J Appl Biobehav Res 5(2):121–143

[22] Cox BJ, Enns MW, Clara IP (2004) Psychological dimensions associated with suicidal ideation and attempts in the National Comorbidity Survey. Suicide Life Threat Behav 34(3):209–219 15385175 10.1521/suli.34.3.209.42781

[23] De Leo D, Padoani W, Scocco P et al (2001) Attempted and completed suicide in older subjects: results from the WHO/EURO multicentre study of suicidal behaviour. Int J Geriatr Psychiatry 16(3):300–310 11288165 10.1002/gps.337

[24] Draper B, Kolves K, De Leo D et al (2014) A controlled study of suicide in middle-aged and older people: personality traits, age, and psychiatric disorders. Suicide Life Threat Behav 44(2):130–138 23952907 10.1111/sltb.12053

[25] Eggert LL, Thompson EA, Herting JR et al (1995) Reducing suicide potential among high-risk youth: tests of a school-based prevention program. Suicide Life Threat Behav 25(2):276–296 7570788

[26] Eggert LL, Thompson EA, Randell BP et al (2002) Preliminary effects of brief school-based prevention approaches for reducing youth suicide–risk behaviors, depression, and drug involvement. J Child Adolesc Psychiatr Nurs 15(2):48–64 12083753 10.1111/j.1744-6171.2002.tb00326.x

[27] Endler NS, Parker JDA (1990) Coping inventory for stressful situations (CISS): manual. Multi-Health Systems, Toronto

[28] Endler NS, Parker JD (1990) Multidimensional assessment of coping: a critical evaluation. J Pers Soc Psychol 58(5):844–854 2348372 10.1037//0022-3514.58.5.844

[29] Eysenck HJ, Eysenck SBG (1975) Manual of the eysenck personality questionnaire. Educational and Industrial Testing Service, San Diego

[30] Eysenck SE, Eysenck HJ, Barrett P (1985) A revised version of the psychoticism scale. Personal Individ Diff 6(1):21–29

[31] Farmer A, Redman K, Harris T et al (2001) The Cardiff sib-pair study: suicidal ideation in depressed and healthy subjects and their siblings. Crisis 22(2):71–73 11727897 10.1027//0227-5910.22.2.71

[32] Fergusson DM, Woodward LJ, Horwood LJ (2000) Risk factors and life processes associated with the onset of suicidal behaviour during adolescence and early adulthood. Psychol Med 30(1):23–39 10722173 10.1017/s003329179900135x

[33] Folkman S, Moskowitz JT (2004) Coping: pitfalls and promise. Annu Rev Psychol 55:745–774 14744233 10.1146/annurev.psych.55.090902.141456

[34] Gairin I, House A, Owens D (2003) Attendance at the accident and emergency department in the year before suicide: retrospective study. Br J Psychiatry 183:28–33 12835240 10.1192/bjp.183.1.28

[35] Gale CR, Deary IJ, Kuh D et al (2010) Neuroticism in adolescence and cognitive function in midlife in the British 1946 birth cohort: the HALCyon program. J Gerontol B Psychol Sci Soc Sci 65B(1):50–56 19864640 10.1093/geronb/gbp082

[36] Ghahramanlou-Holloway M, Bhar SS, Brown GK et al (2012) Changes in problem-solving appraisal after cognitive therapy for the prevention of suicide. Psychol Med 42(6):1185–1193 22008384 10.1017/S0033291711002169

[37] Gibb SJ, Beautrais AL, Fergusson DM (2005) Mortality and further suicidal behaviour after an index suicide attempt: a 10-year study. Aust N Z J Psychiatry 39(1–2):95–100 15660711 10.1080/j.1440-1614.2005.01514.x

[38] Goldberg DP, Hillier VF (1979) A scaled version of the General Health Questionnaire. Psychol Med 9(1):139–145 424481 10.1017/s0033291700021644

[39] Gow AW, Whiteman MC, Pattie A, Deary IJ (2005) Goldberg’s ‘IPIP’ big-five factor markers: internal consistency and concurrent validation in Scotland. Personal Individ Diff 39:317–329

[40] Gunnell D, Magnusson PK, Rasmussen F (2005) Low intelligence test scores in 18 year old men and risk of suicide: cohort study. BMJ 330(7484):167 15615767 10.1136/bmj.38310.473565.8F PMC544986

[41] Haw C, Hawton K, Houston K et al (2001) Psychiatric and personality disorders in deliberate self-harm patients. Br J Psychiatry 178(1):48–54 11136210 10.1192/bjp.178.1.48

[42] Hawton K, Haw C, Casey D et al (2015) Self-harm in Oxford, England: epidemiological and clinical trends, 1996–2010. Soc Psychiatry Psychiatr Epidemiol 50(5):695–704 25488606 10.1007/s00127-014-0990-1

[43] Hawton K, Rodham K, Evans E et al (2002) Deliberate self harm in adolescents: self report survey in schools in England. BMJ 325(7374):1207–1211 12446536 10.1136/bmj.325.7374.1207 PMC135492

[44] Hawton K, Saunders K, Topiwala A et al (2013) Psychiatric disorders in patients presenting to hospital following self-harm: a systematic review. J Affect Disord 151(3):821–830 24091302 10.1016/j.jad.2013.08.020

[45] Higgins JEE, Endler NS (1995) Coping, life stress, and psychological and somatic distress. Eur J Pers 9(4):253–270

[46] Horrocks JH, A House, Owens D (2002) Attendences in the accident and emergency department following self-harm: a descriptive study. University of Leeds, Academic Unit of Psychiatry and Behavioural Sciences

[47] Horwitz AG, Hill RM, King CA (2011) Specific coping behaviors in relation to adolescent depression and suicidal ideation. J Adolesc 34(5):1077–1085 21074841 10.1016/j.adolescence.2010.10.004 PMC3319342

[48] Hubers AAM, Moaddine S, Peersmann SHM et al (2018) Suicidal ideation and subsequent completed suicide in both psychiatric and non-psychiatric populations: a meta-analysis. Epidemiol Psychiatr Sci 27(2):186–198 27989254 10.1017/S2045796016001049 PMC6998965

[49] Jardine R, Martin NG, Henderson AS (1984) Genetic covariation between neuroticism and the symptoms of anxiety and depression. Genet Epidemiol 1(2):89–107 6544237 10.1002/gepi.1370010202

[50] Jarman B, Townsend P, Carstairs V (1991) Deprivation indices. BMJ 303(6801):523 1912874 10.1136/bmj.303.6801.523-a PMC1670803

[51] Jiang GX, Rasmussen F, Wasserman D (1999) Short stature and poor psychological performance: risk factors for attempted suicide among Swedish male conscripts. Acta Psychiatr Scand 100(6):433–440 10626921 10.1111/j.1600-0447.1999.tb10893.x

[52] Kapur N, Cooper J, O’Connor RC et al (2013) Non-suicidal self-injury v. attempted suicide: new diagnosis or false dichotomy? Br J Psychiatry 202(5):326–328 23637107 10.1192/bjp.bp.112.116111

[53] Kendler KS, Gardner CO, Prescott CA (2003) Personality and the experience of environmental adversity. Psychol Med 33(7):1193–1202 14580074 10.1017/s0033291703008298

[54] Kendler KS, Neale MC, Kessler RC et al (1993) A longitudinal twin study of 1-year prevalence of major depression in women. Arch Gen Psychiatry 50(11):843–852 8215810 10.1001/archpsyc.1993.01820230009001

[55] Kessler RAG, Mroczek D, Ustun B, Wittchen HU (1998) The world health organisation composite international diagnostic interview short-form (CIDI-SF). Int J Methods Psychiatric Res 7(4):171–185

[56] Kessler RC, Borges G, Walters EE (1999) Prevalence of and risk factors for lifetime suicide attempts in the National Comorbidity Survey. Arch Gen Psychiatry 56(7):617–626 10401507 10.1001/archpsyc.56.7.617

[57] Lahey BB (2009) Public health significance of neuroticism. Am Psychol 64(4):241–256 19449983 10.1037/a0015309 PMC2792076

[58] Lezak MD (1995) Neuropsychological testing. Oxford University Press, Oxford

[59] Marioni RE, Batty GD, Hayward C et al (2014) Common genetic variants explain the majority of the correlation between height and intelligence: the generation Scotland study. Behav Genet 44(2):91–96 24554214 10.1007/s10519-014-9644-z PMC3938855

[60] Maris RW (2002) Suicide. Lancet 360(9329):319–326 12147388 10.1016/S0140-6736(02)09556-9

[61] Marrs B, Cornish R, Heron J, Boyd A, Crane C, Hawton K, Lewis G, Tilling K, Macleod J, Gunnell D (2016) Using data linkage to investigate inconsistent reporting of self-harm and questionnaire non-response. Arch Suicide Res 20(2):113–141 26789257 10.1080/13811118.2015.1033121 PMC4841016

[62] McCrae RR, Costa PT Jr (1987) Validation of the five-factor model of personality across instruments and observers. J Pers Soc Psychol 52(1):81–90 3820081 10.1037//0022-3514.52.1.81

[63] National Collaborating Centre for Mental Health (2004) Self Harm—The short-term physical and psychological management and secondary prevention of self-harm in primary and secondary care. National Clinical Practice Guideline 21834185

[64] Navrady LB, Adams MJ, Chan SWY et al (2017) Genetic risk of major depressive disorder: the moderating and mediating effects of neuroticism and psychological resilience on clinical and self-reported depression. Psychol Med 48(11):1890–1899 29183409 10.1017/S0033291717003415 PMC6088772

[65] Navrady LB, Ritchie SJ, Chan SWY et al (2017) Intelligence and neuroticism in relation to depression and psychological distress: evidence from two large population cohorts. Eur Psychiatry 43:58–65 28365468 10.1016/j.eurpsy.2016.12.012 PMC5486156

[66] Navrady LB, Wolters MK, MacIntyre DJ et al (2018) Cohort Profile: stratifying Resilience and Depression Longitudinally (STRADL): a questionnaire follow-up of Generation Scotland: Scottish Family Health Study (GS:SFHS). Int J Epidemiol 47(1):13–14g 29040551 10.1093/ije/dyx115 PMC5837716

[67] Nock MK (2010) Self-injury. Annu Rev Clin Psychol 6:339–363 20192787 10.1146/annurev.clinpsy.121208.131258

[68] Nock MK, Borges G, Bromet EJ et al (2008) Cross-national prevalence and risk factors for suicidal ideation, plans and attempts. Br J Psychiatry 192(2):98–105 18245022 10.1192/bjp.bp.107.040113 PMC2259024

[69] Office of National Statistics (2017) Suicides in the UK. https://www.ons.gov.uk/peoplepopulationandcommunity/birthsdeathsandmarriages/deaths/datasets/suicidesintheunitedkingdomreferencetables. Accessed 5 Feb 2019

[70] Olfson M, Wall M, Wang S et al (2017) Suicide following deliberate self-harm. Am J Psychiatry 174(8):765–774 28320225 10.1176/appi.ajp.2017.16111288

[71] Olfson M, Wang S, Blanco C (2015) National trends in hospital-treated self-harm events among middle-aged adults. Gen Hosp Psychiatry 37(6):613–619 26380873 10.1016/j.genhosppsych.2015.08.004

[72] Petronis KR, Samuels JF, Moscicki EK et al (1990) An epidemiologic investigation of potential risk factors for suicide attempts. Soc Psychiatry Psychiatr Epidemiol 25(4):193–199 2399476 10.1007/BF00782961

[73] Pickles A, Aglan A, Collishaw S et al (2010) Predictors of suicidality across the life span: the Isle of Wight study. Psychol Med 40(9):1453–1466 19939326 10.1017/S0033291709991905

[74] Platt SHK (2000) Suicidal behaviour and the labour market. In: Hawton KVHK (ed) Handbook of suicide and attempted suicide. Wiley, Chichester

[75] Rappaport LM, Flint J, Kendler KS (2017) Clarifying the role of neuroticism in suicidal ideation and suicide attempt among women with major depressive disorder. Psychol Med 47(13):2334–2344 28397619 10.1017/S003329171700085X PMC5595639

[76] Schmidtke A, Bille-Brahe U, DeLeo D et al (1996) Attempted suicide in Europe: rates, trends and sociodemographic characteristics of suicide attempters during the period 1989–1992. Results of the WHO/EURO Multicentre Study on Parasuicide. Acta Psychiatr Scand 93(5):327–338 8792901 10.1111/j.1600-0447.1996.tb10656.x

[77] Scottish Government (2009) Scottish index of multiple deprivation 2009 general report. Office of the Chief Statistician, Edinburgh

[78] Sharif F, Parsnia A, Mani A et al (2014) Comparison of personality traits, coping styles, and psychiatric disorders in adult suicidal and non-suicidal individuals. Int J Community Based Nurs Midwifery 2(3):148–156 25349857 PMC4201200

[79] Skegg K (2005) Self-harm. Lancet 366(9495):1471–1483 16243093 10.1016/S0140-6736(05)67600-3

[80] Smith BH, Campbell A, Linksted P et al (2013) Cohort Profile: Generation Scotland: Scottish Family Health Study (GS:SFHS) The study, its participants and their potential for genetic research on health and illness. Int J Epidemiol 42(3):689–700 22786799 10.1093/ije/dys084

[81] Smith BH, Campbell H, Blackwood D et al (2006) Generation Scotland: the Scottish Family Health Study; a new resource for researching genes and heritability. BMC Med Genet 7:74 17014726 10.1186/1471-2350-7-74 PMC1592477

[82] Smith DJ, Nicholl BI, Cullen B et al (2013) Prevalence and characteristics of probable major depression and bipolar disorder within UK biobank: cross-sectional study of 172,751 participants. PLoS One 8(11):e75362 24282498 10.1371/journal.pone.0075362 PMC3839907

[83] Sorberg A, Allebeck P, Melin B et al (2013) Cognitive ability in early adulthood is associated with later suicide and suicide attempt: the role of risk factors over the life course. Psychol Med 43(1):49–60 22617391 10.1017/S0033291712001043

[84] Statham DJ, Heath AC, Madden PA et al (1998) Suicidal behaviour: an epidemiological and genetic study. Psychol Med 28(4):839–855 9723140 10.1017/s0033291798006916

[85] Sudlow C, Gallacher J, Allen N et al (2015) UK biobank: an open access resource for identifying the causes of a wide range of complex diseases of middle and old age. Plos Med 12(3):e1001779 25826379 10.1371/journal.pmed.1001779 PMC4380465

[86] Sugawara N, Yasui-Furukori N, Sasaki G et al (2012) Coping behaviors in relation to depressive symptoms and suicidal ideation among middle-aged workers in Japan. J Affect Disord 142(1–3):264–268 22835844 10.1016/j.jad.2012.05.011

[87] Tanji F, Kakizaki M, Sugawara Y et al (2015) Personality and suicide risk: the impact of economic crisis in Japan. Psychol Med 45(3):559–573 25036366 10.1017/S0033291714001688 PMC4413788

[88] Taylor R, Page A, Morrell S et al (2004) Socio-economic differentials in mental disorders and suicide attempts in Australia. Br J Psychiatry 185:486–493 15572739 10.1192/bjp.185.6.486

[89] Turecki G, Brent DA (2016) Suicide and suicidal behaviour. Lancet 387(10024):1227–1239 26385066 10.1016/S0140-6736(15)00234-2 PMC5319859

[90] University of York (1998) University of York NHS Centre for reviews and dissemination: deliberate self harm and attempted suicide. Effective Health Care University of York, pp 1–12

[91] Wechsler D (1998) Wechsler adult intelligence scale III. Psychological Corporation, London

[92] Wechsler D (1998) Wechsler memory scale III. Psychological Corporation, London

