# Supplementary material for: The role of neuroticism in self-harm and suicidal ideation: results from two UK population-based cohorts
Source: Soc Psychiatry Psychiatr Epidemiol. 2019 May 23;54(12):1505–18. doi: 10.1007/s00127-019-01725-7 (PMC6858388; doi:10.1007/s00127-019-01725-7)
Supplement: Supplementary file 1 — Supplementary material 1 (DOCX 41 kb) [file 127_2019_1725_MOESM1_ESM.docx]

**SUPPLEMENTARY MATERIAL: The Role of Neuroticism in Self-Harm and Suicidal Ideation**

**Table S1: Male and Female Models from GS:SFHS and UKB**

| **GS:SFHS** | **OR Male (95% CI)** | **pFDR** | **Odds Ratios Female (95% CI)** | **pFDR** | **UKB** | **Odds Ratios Male (95% CI)** | **pFDR** | **Odds Ratios Female(95% CI)** | **pFDR** |
| --- | --- | --- | --- | --- | --- | --- | --- | --- | --- |
| Age 18-24 | 0.05(0.01-0.4) | 0.0004(**) | 0.9(0.3-2.7) | 0.94 |  |  |  |  |  |
| Age 25-34 | 1.4(0.5-3.6) | 0.63 | 3.1(1.3-7.9) | 0.01(*) |  |  |  |  |  |
| Age 35-44 | 1.1(0.5-3.0) | 0.77 | 4.3(2.0-10.0) | 0.0004(**) | Age 35-44 | 1.4(0.9-2.2) | 0.15 | 1.3(0.98-1.7) | 0.15 |
| Age 45-54 | 1.2(0.5-2.9) | 0.72 | 2.4(1.2-5.1) | 0.02(*) | Age 45-54 | 1.7(1.3-2.4) | 0.002(*) | 1.3(1.1-1.6) | 0.04(*) |
| Age 55-64 | Ref |  |  |  | Age 55-64 | Ref |  |  |  |
| Age 65-74 | 0.5(0.1-2.0) | 0.46 | 0.2(0.03-0.7) | 0.01(*) | Age 65-74 | 0.6(0.3-1.0) | 0.10(.) | 0.7(0.5-1.0) | 0.13 |
| Age 75+ | 0.03(0.001-1.6) | 0.13 | 0.2(0.02-1.8) | 0.22 |  |  |  |  |  |
| No history of depression | Ref |  |  |  | No history of depression | Ref |  |  |  |
| History of depression (SCID) | 8.2(3.0-25.9) | 0.0004(**) | 9.8(4.3-26.6) | 0.0004(**) | History of depression (self-report) | 15.2(10.0-24.0) | 2.9x10^-33^(***) | 11.3(8.4-15.6) | 1.5x10^-53^(***) |
| EPQ-SF Neuroticism | 1.3(1.1-1.5) | 0.0004(**) | 1.2(1.1-1.4) | 0.0004(**) | EPQ-SF Neuroticism | 1.1(1.1-1.2) | 6.8x10^-07^(***) | 1.1(1.1-1.1) | 7.91x10-^10^(***) |
| Cognitive function (g) | 0.8(0.6-1.0) | 0.17 | 0.7(0.5-0.9) | 0.001(*) | Cognitive function(g) | 1.1(1.0-1.3) | 0.08(.) | 1.1(1.0-1.2) | 0.37 |
| Socioeconomic deprivation quintile (SIMD) | 0.7(0.5-0.9) | 0.01(*) | 0.8(0.6-0.9) | 0.003(*) |  |  |  |  |  |
|  |  |  |  |  | Socioeconomic deprivation (Townsend score) | 1.1(1.1-1.2) | 6.8x10^-7^ | 1.1(1.1-1.1) | 8.61x10^-8^(***) |
| Educational attainment :  No Qualification | 2.0(0.5-8.9) | 0.49 | 2.9(1.0-7.6) | 0.049(*) | Educational attainment :  None of the above | 0.7(0.4-1.4) | 0.41 | 1.1(0.6-1.8)) | 0.72 |
| O Levels/GCSEs or equivalent | 1.5(0.4-5.0) | 0.63 | 1.0(0.4-2.5) | 0.93 | O levels/GCSEs or equivalent | 0.7(0.4-1.1) | 0.16 | 1.1(0.9-1.6) | 0.47 |
|  |  |  |  |  | CSEs or equivalent | 0.8(0.4-1.6) | 0.62 | 1.1(0.7-1.8) | 0.72 |
| A Levels or equivalent | Ref |  |  |  | A Levels | Ref |  |  |  |
| NVQ or equivalent | 1.3(0.5-3.9) | 0.68 | 1.4(0.6-3.3) | 0.42 | NVQ or equivalent | 0.7(0.4-1.3) | 0.35 | 1.5(0.9-2.4) | 0.22 |
| College or University Degree | 0.4(0.1-1.5) | 0.32 | 0.6(0.2-1.4) | 0.31 | College or University degree | 0.6(0.4-0.9) | 0.02(*) | 1.2(0.9-1.6) | 0.24 |
|  |  |  |  |  | Other professional qualifications eg: nursing, teaching | 0.9(0.4-1.8) | 0.73 | 1.0(0.6-1.6) | 0.93 |
| Household relationship status: Couple | Ref |  |  |  | Household relationship status: Couple | Ref |  |  |  |
| Household relationship  status :Single | 3.2(1.5-7.0) | 0.001(**) | 2.1(1.3-3.7) | 0.004(*) | Household relationship  status :Single | 1.6(1.2-2.1) | 0.006(*) | 1.2(1.0-1.4) | 0.24 |

**Table S2 : Correlation Matrix of Variables in Re-Contact Study**

| **Correlation Matrix** | |  |  |  |  |
| --- | --- | --- | --- | --- | --- |
|  |  |  |  |  |  |
|  | Neuroticism | AoC | EoC | ToC | BLEQ Total |
| Neuroticism |  |  |  |  |  |
| AoC | 0.13 |  |  |  |  |
| EoC | 0.50 | 0.02 |  |  |  |
| ToC | -0.18 | 0.20 | -0.18 |  |  |
| BLEQ Total | 0.12 | 0.03 | 0.08 | -0.02 |  |

**List of Threatening Experiences (LTE)**

1. Serious injury or assault to yourself
2. Serious injury or assault to a close relative
3. Death of a parent, spouse, child or sibling
4. Death of a close family friend or other relative
5. Separation due to marital difficulties or break up of a steady relationship
6. Serious problem(s) with close friend, neighbour or relative
7. Made redundant or sacked from job
8. Seeking work unsuccessfully for more than one month
9. Major financial crisis (such as losing three month’s income)
10. Problems with the police involving court appearance
11. Something of value lost or stolen
12. Yourself or your partner give birth

Source : Brugha, T., Bebbington, P., Tennant, C., & Hurry, J. (1985). The List of Threatening Experiences: a subset of 12 life event categories with considerable long-term contextual threat. Psychological Medicine, 15(1), 189-194.

**Table S3: Multivariable analysis of predictors of history of suicidal ideation in GS Re-Contact Study (N=3342)**

|  | **History of suicidal ideation** | **Controls** |  |
| --- | --- | --- | --- |
|  | n=158  (%/s.d.) | N=3184  (%/s.d.) | p value (Effect size) |
|  |  |  |  |
| Female gender  *n* (%) | 84(53.2) | 1913(60.1) | 0.08 |
| Age  *mean*(s.d.) | 53.6(12.7) | 56.2(12.1) | 0.02 (0.21) |
| History of depression(CIDI)  *n* (%) | 76(48.1) | 529(16.6) | <2x 10^-16^  (0.69) |
| EPQ Neuroticism score  *mean*(s.d.) | 5.7(3.4) | 3.3(3.0) | <5.3x 10^-15^  (0.80) |
| Cognitive ability(g)  *mean*(s.d.) | 0.13(1.3) | 0.42(1.2) | 0.005 (0.25) |
| Socioeconomic deprivation (SIMD) rank  *mean*(s.d.) | 3664(1975) | 42198(1743) | 0.0007 (0.32) |
| History of self-harm  *n* (%) | 21 (13.3) | 59 (1.9) | <2.2x 10^-16^  (0.47) |
| List of Threatening Experiences total  *mean*(s.d.) | 1.6(1.6) | 0.9(1.3) | <2.90x 10^-7^  (0.55) |
| CISS Emotion  oriented coping  *mean*(s.d.) | 49.5(12.3) | 36.6(11.9) | <2.2x 10^-16^ (1.0) |
| CISS Task oriented coping  *mean*(s.d.) | 48.5(12.9) | 55.5(11.4) | <2.62x 10^-10^  (0.61) |
| CISS Avoidance oriented coping  *mean*(s.d.) | 38.6(9.2) | 39.4(10.3) | 0.28 |

Effect sizes are shown using Cohen’s *d* (quantitative) and Cohen’s *h* (categorical). Abbreviations : OR = Odds Ratio. 95%CI = 95% Confidence Interval. EPQ = Eysenck Personality Questionnaire-revised Short Form. SIMD = Scottish Index of Multiple Deprivation. CISS = Coping Inventory for Stressful Situations CIDI = Composite International Diagnostic Interview

**TABLE S4.A - Analysis of Complete Case versus Whole Sample proportions and missing data for GS:SFHS and UKB analysis of predictors of history of self-harm requiring hospital attendance**

| **GS:SFHS** | 15798 |  | 20685 |  | **Prop test** | **UKB** |  |  |  |  |  | **Cohens h** |
| --- | --- | --- | --- | --- | --- | --- | --- | --- | --- | --- | --- | --- |
|  | **Complete Cases** | % | **Whole Sample** | **%** | **p** |  | **Complete Cases** | **%** | **Whole Cohort** | **%** | **p** | **Effect size** |
| Male | 6544 | 41.4 | 8489 | 41.0 | 0.46 | Male | 16092 | 45.7 | 22498 | 46.2 | 0.11 |  |
| Female | 9254 | 58.6 | 12196 | 59.0 |  | Female | 19135 | 54.3 | 26164 | 53.8 |  |  |
| 18-24 | 1506 | 9.5 | 1963 | 9.5 | 0.89 | 18-24 | N/A |  | N/A |  |  |  |
| 25-34 | 2115 | 13.4 | 2711 | 13.1 | 0.43 | 25-34 | N/A |  | N/A |  |  |  |
| 35-44 | 2965 | 18.8 | 3836 | 18.5 | 0.59 | 35-44 | 3328 | 9.4 | 4601 | 9.5 | 0.97 |  |
| 45-54 | 3448 | 21.8 | 4603 | 22.3 | 0.33 | 45-54 | 9899 | 28.1 | 13623 | 28.0 | 0.74 |  |
| 55-64 | 4184 | 26.5 | 5313 | 25.7 | 0.09 | 55-64 | 16325 | 46.3 | 22972 | 47.2 | 0.013 | 0.02 |
| 65-74 | 1255 | 7.9 | 1713 | 8.3 | 0.24 | 65-74 | 5675 | 16.1 | 7466 | 15.3 | 0.0026 | 0.02 |
| 75+ | 325 | 2.1 | 546 | 2.6 | 0.0003 | 75+ |  |  | 0 |  |  |  |
| No MDD | 13787 | 87.3 | 17998 | 87.0 | 0.46 | No MDD | 23054 | 65.4 | 30840 | 63.4 |  |  |
| History MDD | 2011 | 12.7 | 2687 | 13.0 |  | History MDD | 12173 | 34.6 | 17822 | 36.6 | 6.5x10-^10^ | 0.04 |
| **Missing Data** |  |  |  |  |  | **Missing Data** |  |  |  |  |  |  |
| SIMD/Townsend |  |  | 1208 | 5.8 |  | SIMD/Townsend |  |  | 66 | 0.1 |  |  |
| EPQ Neuroticism |  |  | 1758 | 8.5 |  | EPQ Neuroticism |  |  | 0 | 0.0 |  |  |
| Living as couple |  |  | 839 | 4.1 |  | Living as couple |  |  | 0 | 0.0 |  |  |
| Education |  |  | 2097 | 10.1 |  | Education |  |  | 101 | 0.2 |  |  |
| Cognitive ability(g) |  |  | 475 | 2.3 |  | Cognitive ability(g) |  |  | 6086 | 12.5 |  |  |

**TABLE S4.B – Analysis of Complete Case versus Whole Sample proportions and missing data for GS:SFHS follow-up analysis of predictors of suicidal ideation**

| **Follow up study on suicidal ideation** | |  |  |  | **Prop test** |
| --- | --- | --- | --- | --- | --- |
|  | **Complete Cases** | **%** | **Whole Cohort** | **%** | **p value** |
| Male | 1399 | 39.9 | 1534 | 38.7 | 0.27 |
| Female | 2104 | 60.1 | 2432 | 61.3 |  |
| 18-24 | 23 | 0.7 | 23 | 0.6 | 0.67 |
| 25-34 | 232 | 6.6 | 247 | 6.2 | 0.49 |
| 35-44 | 355 | 10.1 | 385 | 9.7 | 0.54 |
| 45-54 | 785 | 22.4 | 871 | 22.0 | 0.64 |
| 55-64 | 1221 | 34.9 | 1391 | 35.1 | 0.84 |
| 65-74 | 780 | 22.3 | 913 | 23.0 | 0.44 |
| 75+ | 107 | 3.1 | 136 | 3.4 | 0.36 |
| No MDD | 2829 | 80.8 | 3200 | 80.7 | 0.94 |
| History MDD | 674 | 19.2 | 766 | 19.3 |  |
| No SH | 3410 | 97.3 | 3842 | 96.9 | 0.23 |
| History SH | 93 | 2.7 | 124 | 3.1 |  |
|  |  |  |  |  |  |
|  |  |  |  |  |  |
| **Missing Data** |  |  |  | % |  |
| SIMD/Townsend |  |  | 0 | 0.0 |  |
| EPQ Neuroticism |  |  | 0 | 0.0 |  |
| List of threatening experiences | |  | 10 | 0.3 |  |
| CISS Emotion oriented coping | |  | 144 | 3.6 |  |
| CISS Task oriented coping | |  | 204 | 5.1 |  |
| CISS Avoidance oriented coping | |  | 172 | 4.3 |  |
